# Interventional Prospective Studies on Xerostomia in Patients Undergoing Palliative and End-of-Life Care: A Scoping Review

**DOI:** 10.7759/cureus.63002

**Published:** 2024-06-23

**Authors:** Yasumasa Kakei, Maiko Shimosato, Sakiko Soutome, Madoka Funahara, Yuko Shikama, Yuki Sakamoto, Yumiko Ikegami, Mitsunobu Otsuru, Nagato Natsume, Masahiro Umeda

**Affiliations:** 1 Department of Oral and Maxillofacial Surgery, Kobe University Hospital, Kobe, JPN; 2 Department of Oral and Maxillofacial Surgery, National Hospital Organization Kyoto Medical Center, Kyoto, JPN; 3 Department of Oral Health, Nagasaki University Graduate School of Biomedical Sciences, Nagasaki, JPN; 4 School of Oral Health Sciences, Kyushu Dental University, Kitakyusyu, JPN; 5 Department of Oral and Maxillofacial Surgery, Nagoya City University Hospital, Nagoya, JPN; 6 Department of Oral Surgery, Kansai Medical University Medical Center, Moriguchi, JPN; 7 Department of Nursing, Tokyo Metropolitan Cancer and Infectious Diseases Center Komagome Hospital, Tokyo, JPN; 8 Department of Oral and Maxillofacial Surgery, Kanagawa Dental University, Yokosuka, JPN; 9 Division of Research and Treatment for Oral and Maxillofacial Congenital Anomalies, School of Dentistry, Aichi Gakuin University, Nagoya, JPN; 10 Department of Clinical Oral Oncology, Nagasaki University Graduate School of Biomedical Sciences, Nagasaki, JPN

**Keywords:** oral problem, oral care, prospective interventional study, palliative and end-of-life care, xerostomia

## Abstract

Patients undergoing palliative care often develop debilitating oral conditions, including xerostomia. These conditions may significantly impact patients’ quality of life. Despite the high prevalence and adverse impact of xerostomia, effective management strategies remain unclear. This scoping review was performed to elucidate effective interventions for xerostomia in patients undergoing palliative and end-of-life care. A comprehensive search strategy was employed to identify relevant studies up to August 2023. Full-text primary articles focusing on xerostomia in patients receiving palliative care were included in the review. Eleven articles were selected for analysis, and data were extracted by six reviewers. This review followed the Preferred Reporting Items for Systematic Reviews and Meta-Analyses guidelines. Among the 11 studies, interventions ranged from oral care to saliva substitutes and methods to stimulate saliva secretion. The primary method of assessing xerostomia was the performance of subjective evaluations using visual analog scale scores or numerical rating scale scores. Various interventions including oral care regimens, topical treatments, and mixed efficacy outcomes were reported. Notably, only one study directly measured the saliva volume, highlighting a reliance on subjective endpoints in most studies. Although no definitive conclusions can be drawn regarding the most effective intervention, oral care was a preferred option for managing xerostomia in patients undergoing palliative care. Additionally, adjunctive treatments such as ice cubes, saline, and moisturizers showed promise but require further investigation. Objective measures should be incorporated into future intervention trials to complement subjective assessments and provide a comprehensive evaluation of xerostomia management strategies in this patient population.

## Introduction and background

The deteriorating oral conditions experienced by many patients in palliative cancer care can result in a range of distressing symptoms, including xerostomia (a subjective complaint of dryness in the mouth), orofacial pain, dysphagia, and mucositis [1,2]. These symptoms can significantly impact daily life, affecting eating, communication, and sleep, and are recognized as a major cause of reduced quality of life [3,4]. While xerostomia, mucositis, and dysphagia are all common oral problems of patients undergoing palliative care, xerostomia is the most frequently observed, with prevalence rates ranging from 30% to 88% [5-8]. The most common causes of xerostomia in this patient population are drug therapy, dehydration, and cancer-related cachexia in those with terminal-stage cancer [9-12]. Although medication remains a vital component of palliative care, current guidelines indicate that increasing hydration therapy does not improve xerostomia [13-15]. Especially, at the end of life, various causes of xerostomia coexist and are related to one another. Many drugs used during this period have side effects that cause xerostomia but are difficult to discontinue, resulting in intense xerostomia [16]. Some drugs, such as pilocarpine [17], can be prescribed for xerostomia; however, even if they are effective for palliation of xerostomia at the end of life, there are issues of insurance coverage and the need for more oral medication. Accordingly, the effective management of xerostomia requires the implementation of preventive measures.

In terminally ill patients, xerostomia is often both a sign of disease progression and a medication side effect, which poses a challenge in eliminating the underlying causes during palliative care [1,18,19]. Xerostomia can cause significant discomfort and can impact eating, drinking, and communication abilities [3,4]. While managing xerostomia is essential in patients undergoing palliative therapy and in terminally ill patients, treatment of other diseases and symptoms may take priority over care for palliation of oral symptoms [18,19]. Various treatment approaches have been tried in the past, including artificial saliva [20], saliva stimulants [21], oral care [22], and acupuncture [23], but the most effective intervention remains unclear. The objective of this review is to provide clarity regarding effective strategies for managing xerostomia in patients undergoing palliative and end-of-life care.

## Review

Methodology

The research team designed a search strategy that included two specific databases, English-language publications, and comprehensive search terms to ensure no relevant primary studies were missed. Table 1 provides the detailed data sources.

**Table 1:** Details of data sources.

Databases	PubMed, Cochrane Library
Other resources	Reference lists and a manual search in key journals
Search period	Until August 2023
Language	Primary studies published in English
Search terms	“Xerostomia,” “Palliative”

**Figure 1:**
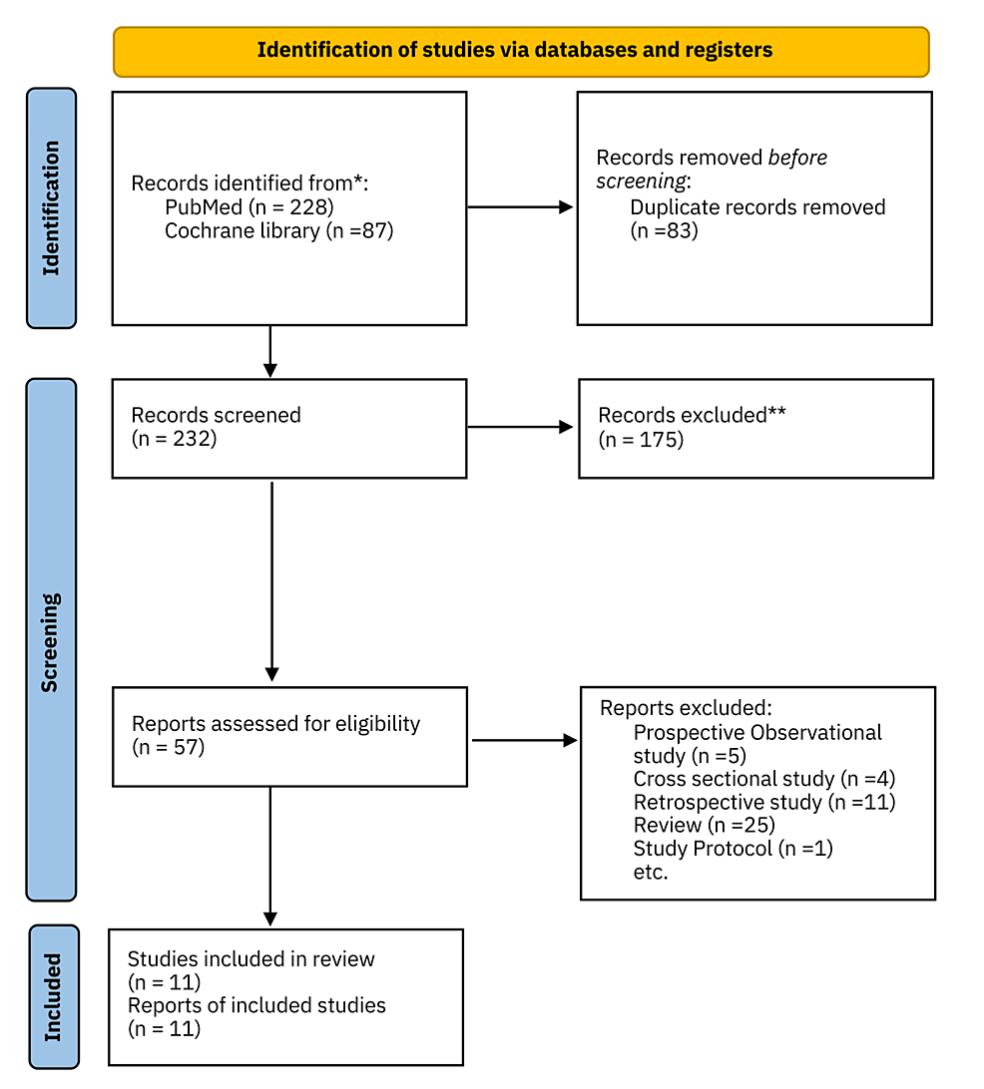
Process of study selection.

The inclusion criteria were full-text primary articles focused on xerostomia in patients undergoing palliative care published until August 2023. The study selection process followed the Preferred Reporting Items for Systematic Reviews and Meta-Analyses (PRISMA), and the PRISMA flowchart is shown in Figure 1.

The initial search identified 315 articles; after the removal of duplicates, 232 articles remained. Screening of relevant abstracts yielded 232 studies, with 57 meeting the inclusion criteria. Of these, 46 were excluded, and a consensus among all reviewers resulted in 11 articles being used for further analysis. Six reviewers (M.F., S.S., M.O., Y.S., Y.S., and M.U.) extracted the following study details and entered them into a table: author(s), years of publication, setting, title, contents of intervention, and study design. The number of enrolled patients undergoing palliative care, number of patients who completed the study, endpoint for xerostomia, time of completion of evaluation items, and results were also extracted and summarized based on the respective research questions.

Results

Literature Review

The search resulted in 232 hits in PubMed and the Cochrane Library. The downloaded full-text articles were read, and 11 articles met the inclusion criteria (Figure 1).

Characteristics of Studies

This review included a total of 11 articles, which are summarized in Table 2 and Table 3.

**Table 2:** Summary of included studies.

Authors/Year/Setting	Reference	Intervention	Study design
Sweeney et al./1997/United Kingdom	[24]	Mucin-containing oral spray (Saliva Orthana)	A randomized, double-blinded, placebo-controlled study
Davies et al./1998/United Kingdom	[20]	Artificial saliva (Saliva Orthana) and pilocarpine	A randomized, open-label, crossover study
Rydholm and Strang/1999/Sweden	[23]	Acupuncture	A single-arm, open-label study
Davies/2000/United Kingdom	[25]	Artificial saliva (Saliva Orthana) and chewing gum	A randomized, open-label, crossover study
Meidell and Holritz Rasmussen/2009/Sweden	[26]	Acupuncture	A single-arm, open-label study
Nikles et al./2015/Australia	[21]	Oral pilocarpine drops	A randomized, double-blinded, crossover study
Davis et al./2017/United States	[27]	Toothpicks containing flavoring with and without jambu extract (spilanthol)	A non-randomized, double-blinded study
Kvalheim et al./2019/Norway	[28]	Three oral moisturizers (glycerol, Aequasyal, and Salient)	A randomized, double-blinded, crossover study
Magnani et al./2019/Italy	[22]	Oral care	A single-arm, open-label study
Monsen et al./2021/Norway	[29]	*Salvia officinalis*-based herbal mouth rinse and normal saline	A single-blinded (by researchers) randomized study
Phelan et al./2023/Australia	[30]	Mini mint ice cubes and plain ice chips	A randomized, open-label, crossover study

**Table 3:** Summary of included studies (continued). NRS = numerical rating scale; EORTC QLQ-OH17 = European Organisation for Research and Treatment of Cancer Quality of Life Questionnaire–Oral Health module with 17 items; VAS = visual analog scale

Authors/Year/Setting	Reference	Endpoint on xerostomia	Time of completion of the evaluation	Completed subjects/Enrolled subjects	Results
Sweeney et al./1997/United Kingdom	[24]	VAS	After 14 days	26/35 (74%)	There were no statistically significant differences in the VAS scores between the mucin-containing spray and placebo spray
Davies et al./1998/United Kingdom	[20]	VAS	After five weeks	26/70 (37%)	Pilocarpine was found to be more effective than artificial saliva in terms of the mean change in VAS scores for xerostomia (p = 0.003)
Rydholm and Strang/1999/Sweden	[23]	VAS	After five weeks	15/20 (75%)	The mean VAS score decreased from 8.8 to 6.1 after five treatments and to 4.0 after the patients had finished all of the treatments, the number of which was 10 for most patients (p < 0.0001)
Davies/2000/United Kingdom	[25]	VAS	After two weeks	26/43 (60%)	Chewing gum scored better than artificial saliva in terms of efficacy. However, none of the results reached statistical significance
Meidell and Holritz Rasmussen/2009/Sweden	[26]	VAS	After five weeks	8/14 (57%)	The VAS score decreased from a median of 7.5 before baseline to 3.3 before the 10th treatment (p = 0.001)
Nikles et al./2015/Australia	[21]	NRS	After 18 days	4/17 (24%)	Four patients completed the treatment; two responded and two did not
Davis et al./2017/United States	[27]	Saliva production in mg/minute	After about two hours	10/10 (100%)	Saliva flow increased by 440% over baseline with the use of a flavored toothpick and 628% over baseline with similarly flavored toothpicks infused with spilanthol; these differences were significant (p = 0.00002)
Kvalheim et al./2019/Norway	[22]	NRS	After three days	75/75 (100%)	Dry mouth intensity was significantly decreased when compared with the recruited patients
Magnani et al./2019/Italy	[28]	Five-point ordinal Likert scale (subjective xerostomia)	After two hours	30/30 (100%)	Of the three products, glycerol provided the best relief from xerostomia directly after application but had no effect after two hours. The effects of Aequasyal and Salient were largely maintained after two hours
Monsen et al./2021/Norway	[29]	EORTC QLQ-OH17 (1-4 scale)	After four days	73/88 (83%)	Normal saline and *Salvia officinalis *rinses resulted in similarly improved oral comfort mean scores on 12 items of the EORTC QLQ-OH17 between study days one and five (p = 0.001 and p = 0.003, respectively). An overall significant difference between the two groups was not detected
Phelan et al./2023/Australia	[30]	NRS	After 48 hours	30/30 (100%)	Mint and plain ice cubes produced improvements in symptoms immediately after interventions. Results from dry mouth ratings showed a decrease of 1.6 points for plain ice cubes (p < 0.0001). On average, ratings for mint ice cubes decreased by 3.7 points (p < 0.0001). The average decrease in dry mouth and thirst intensity scores from pre-intervention to post-intervention was significantly greater for mint ice cubes (p < 0.05)

The interventions used in the studies included oral care [22], saliva substitutes [22,24,25], and various approaches to stimulate saliva secretion (i.e., acupuncture [23,26], ice cubes [30], toothpicks [27], and pilocarpine [21]). Of the 11 studies, seven were randomized controlled trials [20,21,24,25,28-30], and four were non-randomized trials [22,23,26,27]. Three of the seven randomized trials were double-blind [21,24,28], and five of the 11 trials were crossover studies [20,21,25,28,30]. The most common method of assessing xerostomia was subjective evaluation using the visual analog scale (VAS) scores or numerical rating scores. Studies with a duration exceeding one week tended to have lower completion rates, with the most recent report from 2017 showing that many studies were completed within one week, and the completion rate was 100% in four of the five studies. Sweeney et al. [24] conducted the first prospective intervention trial on palliative care patients using Saliva Orthana, a mucin-containing saliva substitute, and reported no difference in symptoms between patients given Saliva Orthana and those given a mucin-free placebo. In two randomized crossover trials of Saliva Orthana versus oral pilocarpine, Davies et al. [20] noted a study completion rate of 37%, which was lower than that reported by Sweeney et al. [24]. However, Davies et al. [20] also reported significantly higher salivary VAS scores in the pilocarpine group compared with Sweeney et al. [24]. In addition, in 2000, Davies [25] conducted a two-group comparison study of artificial saliva and chewing gum, a type of salivary stimulation. No significant difference was noted between the two groups, although the completion rate increased to 60% when the study period was shortened. Nikles et al. [21] conducted a trial of oral pilocarpine drops to reduce the side effects of pilocarpine, which were shown to be effective but also cause discomfort, but only four of 17 patients completed the study, and only those of those were effective. Rydholm and Strang [23] and Meidell et al. [26] reported a high benefit of acupuncture, with an increase in VAS scores after treatment and a high study completion rate; however, both of these were single-arm, open-label studies. Davis et al. [27] tested the efficacy of toothpicks in patients in a palliative care ward who had opioid-induced xerostomia. Unlike other studies, this study directly measured saliva volume, which is a more objective measure; however, it was a pilot study involving only 10 patients. Magnani et al. [22] showed that oral care was effective in improving xerostomia, with a high enrollment completion rate despite having the largest sample size of 75 patients. Kvalheim et al. [28] found that oral moisturizers other than glycerol were effective in improving xerostomia for about two hours. Monsen et al. [29] performed a before-and-after comparison of saline and herbal saline. The authors reported that while both showed significant improvement in xerostomia, there was no clear difference between the two groups. Phelan et al. [30] analyzed a total cohort of 30 patients who received either regular ice cubes (n = 14) or mint-flavored ice cubes (n = 16) and found that the mint ice cubes were more clinically effective.

Discussion

This scoping review aimed to determine effective treatments for xerostomia in patients undergoing palliative and end-of-life care. Our findings showed no clear evidence of efficacy for artificial saliva [24], chewing gum [25], or topical pilocarpine [21]. Acupuncture was considered effective; however, the single-group study and small sample size prevented us from definitively concluding that acupuncture was an effective treatment [23,26]. The results for toothpick treatment were very promising in that the study was double-blinded and evaluated by objective saliva volume; however, this was a pilot study and the sample size was very small (10 cases) for the evaluation of toothpick use in edentulous patients [27]. Therefore, future validation studies would be needed to draw any conclusions. A large, single-group trial of an oral care regimen was considered to be an effective treatment [22]. During oral care, moisturizers such as oxygenated glycerol triesters are generally used for patients with xerostomia. Kvalheim et al. concluded that two such moisturizers were superior to glycerin [28]. However, the moisturizer trial was not suitable for a meta-analysis because of differences in endpoints and effects. In parallel with oral care, not only moisturizers but also saline solution and ice cubes can be used. The improvement of xerostomia with saline was shown to be highly beneficial in the study by Monsen et al. [29], although no significant difference was demonstrated between saline with or without herbs. In addition, Phelan et al. [30] reported the effectiveness of plain ice cubes as well as ice cubes with mint. The addition of these interventions to oral care could improve xerostomia in palliative care patients. Although our review has the limitation of being a scoping review rather than a systematic review, further evidence could be accumulated in a prospective randomized controlled trial.

In 10 of the 11 studies, the endpoints related to xerostomia were subjective (VAS score and numerical rating scale score); only one study confirmed the saliva volume. Shimosato et al. [31] reported a gap between subjective dryness symptoms and objective measures of oral wetness. In addition, the subjective complaints of dryness by patients with terminal cancer undergoing palliative care may have limitations in terms of measurement. Therefore, future intervention trials may need to include objective assessments (such as assessing hyposalivation) in addition to subjective assessments.

Our results suggest that oral care is likely to be important in improving xerostomia in patients undergoing palliative care. Moisturizers are often administered after oral care, but more research is needed to determine the optimal moisturizer because the ones that are currently available have drawbacks.

## Conclusions

Previous intervention studies have shown a preference for oral care over other types of interventions for palliation of xerostomia. In addition to oral care, ice cubes, saline, and moisturizers may further reduce xerostomia, but further intervention studies in each setting are needed to obtain more details. To complement the subjective assessments and provide a comprehensive evaluation of xerostomia management strategies in this patient population, future intervention trials should evaluate objective measurements such as hyposalivation.
